# Parental Attitudes towards Child Oral Health and Their Structural Analysis

**DOI:** 10.3390/children11030333

**Published:** 2024-03-11

**Authors:** Apolinaras Zaborskis, Jaunė Razmienė, Augustė Razmaitė, Vilija Andruškevičienė, Julija Narbutaitė, Eglė Aida Bendoraitienė, Aistė Kavaliauskienė

**Affiliations:** 1 Department of Preventive Medicine, Health Research Institute, Faculty of Public Health, Medical Academy, Lithuanian University of Health Sciences, A. Mickevičiaus 9, LT-44307 Kaunas, Lithuania; 2 Department of Oral Health and Paediatric Dentistry, Faculty of Odontology, Medical Academy, Lithuanian University of Health Sciences, A. Mickevičiaus 9, LT-44307 Kaunas, Lithuania; jaune.razmiene@lsmu.lt (J.R.); augurazm0210@lsmu.lt (A.R.); vilija.andruskeviciene@lsmu.lt (V.A.); julija.narbutaite@lsmu.lt (J.N.); egleaida.bendoraitiene@lsmu.lt (E.A.B.); 3 Department of Orthodontics, Faculty of Odontology, Medical Academy, Lithuanian University of Health Sciences, A. Mickevičiaus 9, LT-44307 Kaunas, Lithuania; aiste.kavaliauskiene@lsmu.lt

**Keywords:** children, oral health, tooth brushing, parental attitudes, scales, structural analysis, second-order factor model

## Abstract

The relationship between parental attitudes towards health and child development has been a topic of interest for many years; however, research results in this field are still inconsistent. This study aimed to develop a structural equation model of the Parental Attitudes toward Child Oral Health (PACOH) scale, using this model to analyse the relationship between parental attitudes with demographic variables and the oral health-related behaviour of parents and children. A total of 302 parents (87% mothers) answered questions regarding their own and their children’s, aged 4–7 years, oral health-related actions and completed the 38-item PACOH scale. The structural equation model indicated that parental attitudes captured by the PACOH scale can be fitted to a second-order factorial model, even with the scale shortened to 21 items. The model demonstrated good fit characteristics (CFI = 0.925; IFI = 0.927; GFI = 0.915; RMSEA = 0.049), making it a reliable tool for examining the structure of parental attitudes. This model was employed in the multi-group analysis, revealing the close relationship between positive parents’ attitudes towards their child’s oral health and oral health-promoting behaviour both in parents and children, such as regular tooth brushing (*p* < 0.001), visiting the dentist (*p* = 0.027), and parents helping their child brush his/her teeth (*p* < 0.001). In light of these findings, it was concluded that Parental Attitudes towards Child Oral Health should be considered an essential factor influencing the development of oral health-promoting behaviour in children.

## 1. Introduction

Early childhood, sometimes known as the preschool years, is a crucial developmental stage of life characterised by critical advances in physical, social, cognitive, emotional, linguistic, and parental help domains [1]. During this phase, individuals require habits and behaviours that can have a significant impact on their long-term outcomes [2]. Less than twice-daily tooth brushing and sugar snacking between meals can be considered suitable examples of such kinds of behaviour [3,4,5,6].

There is evidence that poor oral hygiene in children due to neglected tooth brushing facilitates the further development of dental caries [7]. The deciduous teeth should be brushed as carefully as the permanent teeth—twice a day. Preschool-age children, being such young individuals, are not able to brush their teeth properly. Therefore, the parents are responsible for their children’s oral hygiene and help them to brush their teeth regularly [5]. A number of studies have also demonstrated that children are more likely to be caries-free if they grow up in a family in which the frequency of sugar intake is controlled [7,8,9,10]. It is especially important that parents are obliged to control sugar consumption between the meals of their children, as it is followed by a repeated pH drop in saliva, causing the demineralisation of the hard tissues of the tooth [11,12,13]. In addition to the factors already mentioned, a late first visit or irregular visits to the dentist may contribute to dental caries onset [14]. Therefore, parents may be willing to control those risk factors through knowledge and a positive attitude toward the prevention of dental caries in children.

Evidence demonstrates the importance of the family environment in ensuring a good oral and general health state of children. Ultimately, parents are responsible for children’s values, skills, socialisation, and security [15,16]. A number of studies have demonstrated the relationship between parents’ knowledge and attitudes toward children’s oral health with oral health-related behaviours [3,4,5,6,17,18,19,20,21]. Different instruments have been designed and used in previous cross-sectional studies to measure the level of parents’/caregivers’ knowledge, beliefs, and attitudes regarding preschool children’s oral health and hygiene habits; unfortunately, the results of these studies are difficult to compare due to the variety and extent of scales used [22]. Although some scales have been validated, social and cultural differences are calling for the development and validation of more specific scales for use in preschool children. Thus, the search for a suitable scale in this field of studies is still in progress [23].

The scale/questionnaire developed by Pine and Adair et al. (2004) in a multicentre study is one of such attempts that may receive particular attention [21,24]. According to the authors, the items of this instrument are based on psychological models such as the Theory of Planned Behaviour [25], the Health Belief Model [26], and the Health Locus of Control model [27]. Such an approach has the advantage of evaluating the construct of attitudes not only in one dimension but also incorporating other intrinsic factors and thus modifying inappropriate health behaviours [28]. It is a wide-range questionnaire, with items associated with parental beliefs and attitudes to children’s tooth brushing, sugar snacking, and dental caries; consequently, they were divided into the corresponding three dimensions/factors: child tooth brushing, child sugar snaking, and child dental decay [21]. Given the broad scope of this scale, we named it “Parental Attitudes towards Child Oral Health” (PACOH). This scale had previously been used in a multicentre study [19], as well as in national studies [20,29,30]. Some studies have used separate parts of this scale, for example, parents’ attitudes towards children’s tooth brushing [31,32].

In Lithuania, the PACOH scale was translated into Lithuanian, validated, and applied to analyse associations between parental attitudes and possibilities to control oral health behaviour in their children [20,33]. This recent study, like the others [21,24], needs a general analysis of the overall structure of the instrument. The need to use many questions in the questionnaire, as some questions overlapped each other, was reviewed. We hypothesised that the scale could be shortened by the number of items, thereby improving its properties. Following these premises, we aimed to develop a structural equation model of the Parental Attitudes toward Child Oral Health (PACOH) scale, using this model to analyse the relationship of parental attitudes with demographic variables and the oral health-related behaviour of parents and children.

## 2. Materials and Methods

### 2.1. Setting

This study was conducted in the Kaunas region (Kaunas city and the surrounding rural area), Lithuania, in April–June 2023. According to demographic statistics, about 400,000 (2023) inhabitants live in this region, which is 14% of the country’s population [34]. Of these, about 13,000 children aged 4–6 years live in this region, which accounts for 15% of the country; almost all of them attend kindergarten [35]. Among other regions of Lithuania, the Kaunas region is characterised by a high level of industry and a large number of universities and colleges.

### 2.2. Study Design and Sample Size

A cross-sectional study design was used. The sample size was estimated to ensure the reliability and validity of the results from the confirmatory factor analysis (CFA), which is used in this study. Tabachnic and Fidell [36] recommend a minimum sample size of 200–300 for CFA as a rough guideline. These authors also suggest that the sample size can vary depending on the complexity of the model, the number of observed variables, the number of extracted factors, and other reasons; therefore, we chose a sample size of 300.

### 2.3. Subjects

The required number of parents was drawn from lists of children who attended six randomly selected kindergartens located in the Kaunas region (three from Kaunas city and three from the surrounding rural area). Parents (mothers or fathers) of 4–6 year-old children were invited to participate in the current study. Since nearly 30% of parents could refuse to participate in the study [33], we decided to distribute the questionnaires to 425 parents. A total of 307 parents participated in the survey; the response rate was 71%. After excluding questionnaires that were incompletely filled in, 302 questionnaires were included in the analysis.

### 2.4. Questionnaire

The self-administered anonymous questionnaire was filled in by either the father or the mother.

The first section of the questionnaire enquired questions about the respondent’s gender (father or mother), age, education level (whether he/she has graduated from university or college), household location (city or rural area), as well as the child’s gender and age. The respondent was also asked about his/her child’s oral health, tooth brushing habits, and dental visits.

The second section of the questionnaire included 38 statements about parents’ attitudes towards child dental health. These were items from the PACOH scale, which was taken from the study by Adair et al. (2004) [21] (a full list of statements is presented in Section 3). Following this study, the statements comprised three dimensions. The first dimension combined 14 items that expressed parental attitudes towards children’s tooth brushing, including the importance and intention to brush the child’s teeth (b3: “The people in my family would feel it was important to help brush our child’s teeth twice a day”), parental efficacy in relation to child tooth brushing (b7: “I don’t know how to brush my child’s teeth properly”), and parental attitudes towards prevention (b13: “If we brush our child’s teeth twice a day, we can prevent our child getting tooth decay in the future”). The second dimension combined 9 items that expressed the parental attitude towards child sugar snacking, including the importance and intention to control child sugar snacking (s2: “As a family, we intend controlling how often our child has sugary foods or drinks between meals”), and parental efficacy in relation to controlling child sugar snacking (s6: “As a family, we feel it is difficult for us to stop our child having sugary foods”). The third dimension combined 15 items that expressed the parental attitude towards child dental decay, including the perceived seriousness of tooth decay in children (d3: “Tooth decay would have major consequences on our child’s general health”), chance control—decay occurs by chance (d10: “If our child gets tooth decay, it is by chance”), and external control (d15: “The dentist is the best person to prevent tooth decay in our child”). In the paper, we denote these three dimensions as TB, SS, and DD, respectively.

The responses to each item of the PACOH scale were rated on a 4-point Likert scale, giving the following item scores: 1 = ‘strongly agree’; 2 = ‘agree’; 3 = ‘disagree’; 4 = ‘strongly disagree’. If none of these response categories were selected, the item was assigned a value of 2.5. Agreeing with some statements expressed a positive attitude towards children’s dental health (for example, b7: “I don’t know how to brush my child’s teeth properly”), and the values of answers to these statements were inverted. Then, for each PACOH dimension, we summed up the answer scores. Higher sum scores indicated the more positive level of respondent’s attitude.

We used the Lithuanian version of the PACOH scale, which was translated from English, validated, and applied in previous studies [20,33]. Prior to starting the survey, the scale was once again retranslated back into English by a professional. The retranslated version coincided with the original English version, and the meanings of the scale items were retained accurately. The psychometric characteristics of the scale are presented in the Section 3.

### 2.5. Data Analysis

First, descriptive statistics were employed to summarise the characteristics of the sample. Respondents’ responses were estimated by frequencies (n) and percentages (%) and then by the means and asymmetries (skewness). To describe sum scores, we used means and standard deviations. Pearson’s correlation test was utilised to examine the relationship between observed variables, factor values, and sum scores. To assess the differences in sum scores between the two respondents’ groups, Student’s *t*-test was employed as appropriate. Statistical significance was set at *p* < 0.05. Cronbach’s alpha was used to evaluate the internal consistency of each dimension; alpha > 0.6 was considered adequate internal consistency [37]. These analyses were performed using the SPSS statistical package (version 21; IBM SPSS Inc., Chicago, IL, USA).

Next, the structural equation model (SEM) was created using AMOS 21 (IBM SPSS Inc., Chicago, IL, USA, 2012) [38]. The basis of the construction was the model proposed by Adair et al. [19], which consisted of 3 dimensions divided into separate factors. Therefore, the second-order factor analysis approach [39,40] had to be used when constructing the structural model. In the initial structural model, three second-order common factors, TB, SS, and DD, combined 3, 2, and 3 first-order common factors, respectively. The common factors TB, SS, and DD were allowed to be correlated. Parameter estimation was carried out using the maximum likelihood estimate.

Improving the quality of the model was carried out by consistently reducing the number of observed variables from the model. At each step, one observed variable that had the lowest factor loading or the lowest statistical significance was removed from the model. After recalculating the model parameters, the variable removal procedures were repeated. This procedure was stopped as soon as satisfactory model quality was achieved. The use of modification indices helped to identify significant relationships between variables and to increase the quality of the model. The detailed procedure and the path diagram of the final structural model are presented in the Results.

Once the model was calculated with the general sample, a multi-group factor analysis with AMOS was carried out to test the hypothesis about the uniformity of models in different patient groups. Above all, we aimed to check whether the structural means and covariances (the means of the second-order factors and correlation between them) differed between the study groups. Conventionally, the common factor analysis model makes no assumptions about the means of the common factors; however, the option of simultaneous factor analysis for several groups allowed differences in factor means to be estimated across subjects’ groups under reasonable assumptions. These assumptions included model constraints for equal measurement weights and intercepts in the equation for predicting observed variables between groups. To estimate the difference in factor means, the factor means of a control group were fixed to 0 (restricted model), and then the constraints on the factor means of the remaining groups were removed (non-restricted model). Subsequently, the chi-square coefficient of the restricted and non-restricted models was compared; if this coefficient was significantly greater in the restricted model, invariance between the groups could be assumed [38]. The estimate of factor means and correlations were compared with analogous sum score estimates.

The quality of the model was checked by assessing its fit to the survey data. The χ^2^ statistic in relation to the degree of freedom (χ^2^/df) was used to assess the magnitude of the discrepancy between the sample and fitted covariance matrix, where χ^2^/df < 3 indicates that the model and data were consistent [36]. The model fit was also evaluated using the root mean square error of approximation (RSMEA) and other goodness-of-fit statistics as follows: the comparative fit index (CFI), the incremental fit index (IFI), and the goodness of fit index (GFI). The general consensus is that a smaller RMSEA (lower than 0.06) and larger CFI, IFI, and GFI (higher than 0.8) indicate a good fit for real data [41,42,43].

## 3. Results

### 3.1. Sample Characteristics

Table 1 shows the socio-demographic and oral health characteristics of respondents and their children. In addition to these characteristics, significant associations were found between several selected variables. For example, children were more likely to brush their teeth regularly if their parents also did so (r = 0.355; *p* < 0.001); parents reported good child oral health when they helped the child brush his/her teeth (r = 0.277; *p* < 0.001); parents with higher or university education more often indicated that they helped their child brush his/her teeth (r = 0.233; *p* < 0.001).

### 3.2. Analysis of the Original 38-Item Scale

Table 2 presents descriptive statistics (means and assessments of asymmetry) for all 38 items. The percentage of definite answers (‘strongly agree’, ‘agree’, ‘disagree’, ‘strongly disagree’) to scale statements ranged from 77.2% (item d8) to 99.3%. A large asymmetry of respondents’ answers was observed for some statements, in which case the majority of respondents either agreed with the statement (for example, items b1, d5, d7) or disagreed (for example, items b9, b11, d10). The asymmetry in the frequency of answers determined the mean values of the item codes; mostly, it was <2.5 when skewness was >0, and, conversely, it was >2.5 when skewness was <0. In all three original dimensions, internal consistency defined by Cronbach’s alpha was slightly higher than 0.6.

**Figure 1:**
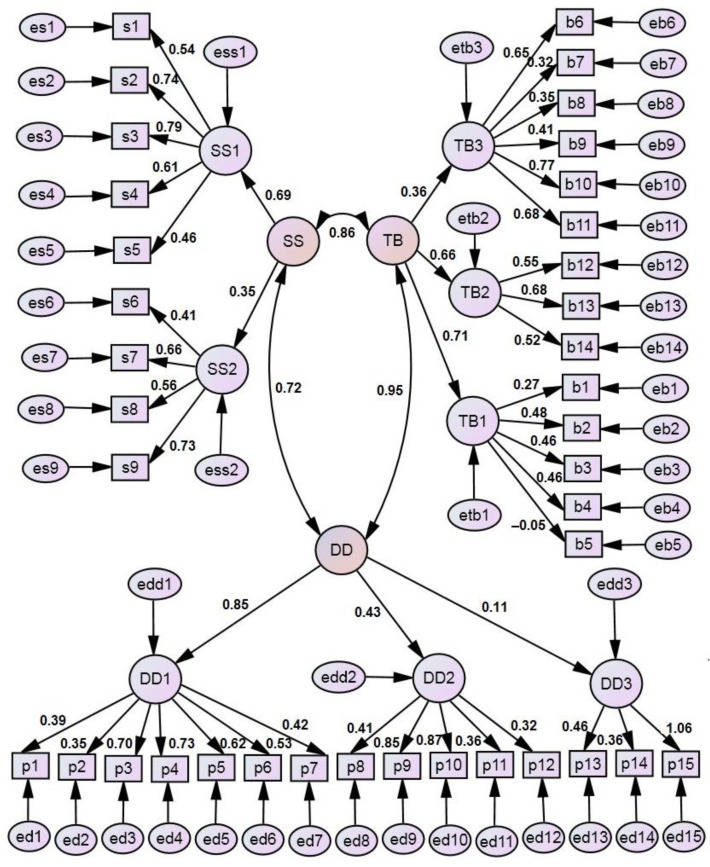
Path diagram of the structural model of the original 38-item scale of Parental Attitude towards Child Oral Health. Dimensions (TB: Parental attitudes towards child tooth brushing behaviour; SS: Parental attitudes towards child sugar snacking; DD: Parental attitudes towards child dental decay) are second-order factors; TB1, TB2, TB3, SS1, SS2, DD1, DD2, and DD3 are first-order factors; rectangles symbolise measured variables (respondents’ opinions about the statements presented in the questionnaire); and ellipses symbolise structural and measurement residuals. The path coefficients leading from the second-order factors to the first-order factors are called structural weights, while the path coefficients leading from the first-order factors to the measured variables are called measurement weights. Correlation coefficients between dimensions are placed next to the two end arcs.

Table 3 shows the factorial structure of the dimensions TB, SS, and DD obtained from the exploratory factor analysis of each dimension. This analysis approved the factorial structure of the dimensions described by Adair et al. [21] with few exceptions arising from items b5 and b8. These results provide the basis for creating a second-order factorial model for the original 38-item PACOH scale (items b5 and b8 were assigned to factors TB3 and TB1, respectively). Figure 1 presents the path diagram of this model. As can be seen from the figure, factor DD3 (external control—preventing decay is the dentist’s responsibility) has a very low weight in the composition of dimension DD; it is worth removing from further analysis. The model fit characteristics of this model were as follows: χ^2^/df = 2.212 < 3; CFI = 0.716; IFI = 0.720; GFI = 0.793; RMSEA = 0.063 < 0.08 (90% CI: 0.059; 0.68); thus, only two characteristics met the good fit criteria.

### 3.3. Shortening the Scale

**Figure 2:**
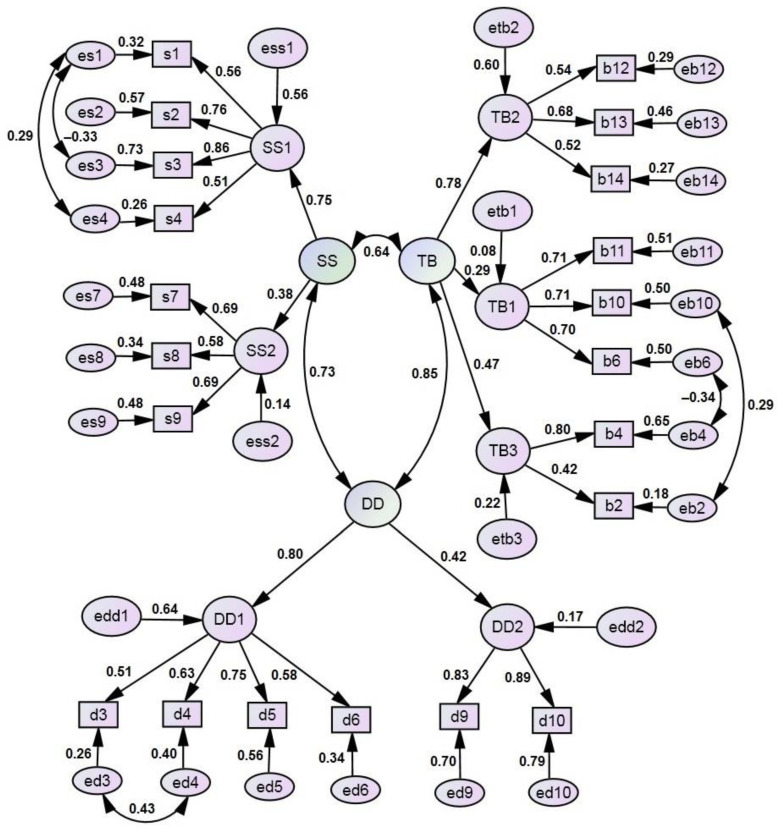
Path diagram of the structural model of the shortened (21-item) scale of Parental Attitudes towards Child Oral Health. Dimension TB: Parental attitudes towards child tooth brushing behaviour; Dimension SS: Parental attitudes towards child sugar snacking; Dimension DD: Parental attitudes towards child dental decay.

To improve the psychometric characteristics of the 38-item scale, 17 items with the lowest weights were removed from the second-order factorial structure in the following sequence: all variables of the factor DD3, then b5, b1, …, s6, d7. Twenty-one items remained in the abbreviated scale: 8 items in the dimension TB, 7 items in the dimension SS, and 6 items in the dimension DD. The use of modification indices helped to identify significant positive correlations between the measurement residuals within dimensions TB, SS, and DD, which significantly improved the fit characteristics of the model. The structural weights varied from 0.290 (Factor TB1: importance and intention to brush child’s teeth) to 0.800 (Factor DD1: perceived seriousness of tooth decay in children). Measurement weights (except for variable b2) were higher than 0.5. In this model, significant (*p* < 0.001) correlations were found between the dimensions TB and DD (r = 0.847), SS and DD (r = 0.732), and TB and SS (r = 0.642). All indices of model fit to empirical data met good criteria suggested by scholars as follows: χ^2^/df = 1.720 < 3; CFI = 0.925 < 0.9; IFI = 0.927 < 0.9; GFI = 0.915 < 0.9; RMSEA = 0.049 < 0.08 (90% CI: 0.039; 0.58). The internal consistency (Cronbach’s alpha) of shortened dimensions was 0.612, 0.689, and 0.725 in dimensions TB, SS, and DD, respectively, which remained similar to those of the original scale. Figure 2 shows the path diagram of the factorial structure of the shortened scale.

### 3.4. Multi-Group Analysis

The purpose of this analysis was to compare parental attitudes between different groups of parents or their children. The means of latent variables (factors/dimensions TB, SS, and DD), correlations between factors, and other explicit model parameters were compared. Although it was not possible to estimate the factor means for all groups, by fixing the factor means of a single group as constant, for instance, to 0, it was possible to obtain meaningful estimates of the factor means for the other groups, i.e., it was possible to determine the difference between the factor means.

In this analysis, we examined 12 pairs of models in which one model of the pair could be obtained by constraining the parameters of the other. The model fit characteristics ranged as follows: χ^2^/df: from 1.308 to 1.588 < 3; CFI: from 0.859 to 0.921; TLI: from 0.849 to 0.915; IFI: from 0.862 to 0.923; RMSEA: from 0.023 to 0.044 < 0.8. For all comparisons, the characteristics χ^2^/df and RMSEA suggested a good model fit to the data presented, while several pairs of models provided an acceptable model fit by CFI, TLI, and IFI.

Table 4 shows the results of the multi-group analysis, presenting differences in factor means and correlations between factors. A comparison of the study groups according to parents’ gender and age showed no significant differences in the average of dimensions TB, SS, and DD. Alternatively, no differences in this model parameter were found when comparing groups of children according to their gender and age. Meanwhile, significantly higher means of one or two dimensions were identified among parents with higher education, parents and children who regularly brushed their teeth and regularly visited the dentist, and those parents who helped their child brush their teeth. Significantly higher means of dimensions were also found in the responses of parents who reported the good status of their teeth (DD dimension) and in the responses of parents from urban areas (SS dimension). A significant correlation between the dimensions TB, SS, and DD existed among most of the comparison groups; however, the values of the correlations varied between the comparison groups. The chi-square coefficients, which were contrasted (Δχ^2^) to test the invariance of factor means and correlations, confirmed the assumption about the difference in estimates between the respondent groups being compared.

The results of the multi-group factor analysis were confirmed by analogous estimates from the sum score analysis. These estimates are presented next to the results of the multi-group factor analysis in Table 4, so the results of both analyses can be compared here. There are only a few exceptions where the results of the sum score analysis contradict the results of the multi-group factor analysis. Thus, considering the variability of factor means and correlations between factors when comparing different groups of study participants, it can be concluded that the shortened PACOH scale retains the properties of discriminant validity, especially when comparing the groups of respondents who differed in terms of the oral health care provided to children.

## 4. Discussion

The present study focuses on parental attitudes toward children’s oral health by analysing the structural properties of the PACOH scale in a Lithuanian sample of respondents. It demonstrated that parental attitudes according to the PACOH scale can be fitted to a second-order factorial model, even if the scale is shortened. The model revealed that parents’ attitudes towards their child’s oral health were significantly associated with positive oral health-related behaviours in both parents and their children. This finding emphasises the importance of parental attitudes towards oral health in forming positive oral health-related behaviours in children.

This study’s methodological background is significant and provides strengths to the research. This was addressed by developing a valid and reliable psychometric measure. The original version of the instrument to measure parental attitudes toward children’s oral health was proposed by Adair et al. (2004) [21]. It comprises 38 items divided into three dimensions, and each of these dimensions consists of several factors. However, examining individual components of the attitude provides a less comprehensive understanding of the characteristics and behaviours of a particular population or subjects than it would be achieved if all the components lined up together [44]. To overcome this limitation, we applied an SEM approach. The use of the second-order factorial model [39,40] allowed us to examine the whole PACOH scale, covering the complex relationships between its components. Unfortunately, some items of the entire 38-item scale undermined the quality of the structural model and were omitted. The structural model of the shortened 21-item PACOH scale resulted in a good fit to empirical data and was effective for multi-group analysis.

In line with the Theory of Planned Behaviour [25], stating that attitudes and actions affect each other, our main study objective was to investigate the relationship between parental attitudes toward their child’s oral health and respondents’ socio-demographic data, oral health status, and oral health-related behaviour. Multi-group analysis with the structural model of the PACOH scale evaluated these relationships, determined which dimensions of the scale were most responsible for these relationships, and assessed whether the structural relationships of the model changed depending on the subject characteristics.

Different age mothers and fathers participated in our study. They answered questions related to the children of both sexes and various ages. Therefore, it was possible to examine whether parental attitudes are related to demographic factors. Multi-group analysis, as well as sum score analysis, revealed that parents’ attitudes towards their children’s oral health were not associated with age and gender in any dimension. This finding is noteworthy from a social psychology perspective, as many studies, including those in health, suggest that gender and age may moderate attitudes [45]. However, our results demonstrate that respondents with a higher education level showed a more positive attitude towards their children’s oral health regarding dental caries prevention, while urban respondents were more likely to control their children’s sugar consumption compared to rural respondents. This is in line with findings from a literature review, indicating that individuals’ attitudes vary based on their level of education and urban/rural residence, which may directly impact attitudes or indirectly influence them through factors such as occupation, income, access to information, healthcare infrastructure and social networks [46]. Overall, understanding how demographic factors correlate to parental attitudes toward oral health is crucial for designing targeted interventions and education programs aimed at improving oral health outcomes, particularly among vulnerable populations, such as young children [47]. By addressing disparities in access to information and resources, healthcare infrastructure, and cultural beliefs, oral health initiatives can better support parents in promoting good oral hygiene practices for their children, regardless of their demographic background [48,49].

This study’s findings demonstrate that parents’ understanding of the importance of brushing their child’s teeth and its efficacy in controlling sugar consumption is higher in the group of children regularly (2 times a day) brushing their teeth. The groups of parents who assisted their child with brushing his/her teeth or whose child visited the dentist regularly had significantly higher levels of parental attitudes. Additionally, parents with good dental health were significantly more likely to understand the importance of preventing tooth decay in their children. The same, but less significant, relationship was observed regarding children’s dental health status. These findings are in line with other studies demonstrating the important role of parental attitudes towards oral health in forming positive health-related behaviour in children [20,21,29,30]. Essentially, parental attitudes predict preventive measures against caries for preschool children [29]. During early childhood, parental supervision and the development of tooth brushing skills, followed by the control of sugar consumption, are the most effective measures in dental caries prevention [3,4,5,6]. Therefore, our study, in line with the theoretical model developed from the Theory of Planned Behaviour [25], provides evidence that changing the attitude of parents can change not only the behaviour of the parents but also the behaviour of children.

This study also has some limitations. On the one hand, some limitations are common for survey studies. First, the cross-sectional nature of this study limits our ability to draw causal inferences. Longitudinal studies are beneficial to understand the dynamics of relationships between parental attitudes and child oral health over time and identify causality in this relationship. Second, our study relies solely on self-reported data, which may be susceptible to recall and social desirability biases. On the other hand, there are specific limitations to our study. First, our study did not aim to determine strategies for improving parental attitudes. Integrating research on attitudes and habits can help researchers determine when and how attitude-directed interventions are most effective in behaviour change strategies [50,51,52]. Second, our study did not include in the questionnaire a section on the child’s diet. We could not compare parental attitudes between groups of children based on the frequency of sugar consumption. In line with previous studies [20,21], it is likely that such groups may differ according to the SS component of parental attitudes.

In summary, our study emphasises the importance of parental attitudes as a significant factor in forming children’s oral health behaviours. It not only contributes to existing knowledge but also calls for further research. Further studies are warranted to delve into the dynamics of relationships between parental attitudes towards oral health and oral health-related behaviour in children. Studies with parental attitude-directed interventions are also recommended. Based on the findings of this study, it can be assumed that such interventions can increase parents’ awareness of the importance of developing their children’s oral hygiene skills. Finally, the multi-group analysis showed that several groups of respondents differed in one or two dimensions of parental attitudes but not in the whole PACOH scale. To clarify the mechanism of such relations, more detailed studies might be also relevant. We are convinced that the short-scale structural model is a reliable tool that can be applied in such research as well as in the oral healthcare practice of preschool children.

## 5. Conclusions

The current study demonstrates that parental attitudes, measured using the PACOH scale, can be effectively fitted to a second-order factorial model, even if the scale is shortened. This model highlighted a close relationship between parents’ positive attitudes toward their child’s oral health and the adoption of oral health-promoting behaviour by both parents and children. These behaviours include regular tooth brushing, visiting the dentist, and parents helping their children to brush their teeth.

These findings emphasise the significance of recognising parental attitudes as a crucial factor affecting the development of positive oral health-related behaviour in children. The implication is that understanding and addressing parental attitudes can be essential for promoting good oral health skills in the younger population.

This study implies a need for further research to investigate whether interventions aimed at modifying or improving parental attitudes can be effective in fostering positive oral health behaviours in children.

## Figures and Tables

**Table 1:** Socio-demographic and oral health characteristics of respondents and their children.

Characteristic	n	%
Gender of respondents:		
males (fathers)	40	13.2
females (mothers)	262	86.8
Age of respondents:		
<35 years	165	54.6
≥35 years	137	45.4
Gender of children:		
boys	164	54.3
girls	138	45.7
Age of children:		
<5 years	151	50.0
≥5 years	151	50.0
Kindergartens location:		
urban area	202	66.9
rural area	100	33.1
Education level of respondents:		
less than college or university	76	25.2
college or university	226	74.8
The respondent brushes his/her teeth:		
2 times a day	228	75.5
less than 2 times a day	74	24.5
The child brushes his/her teeth:		
2 times a day	167	55.3
less than 2 times a day	135	44.7
How does the child brush his/her teeth:		
the child brushes his teeth by himself	142	47.0
parents help the child brush his/her teeth	160	53.0
The respondent visits the dentist with the child:		
once a year or more frequently	187	61.9
irregularly	115	38.1
Respondent’s dental health status:		
good	244	80.8
poor	58	19.2
Child’s dental health status:		
good	240	79.5
poor	62	20.5

**Table 2:** Dimensions, factors and items of original 38-item scale of Parental Attitudes towards Child Dental Health and several statistical estimations of its items.

Item No.	Dimensions, Factors, and Items	Attitude ^1^	Percentage of Definite Answers ^2^	Item Mean ^3^	Skewness
***Dimension TB: Parental attitudes towards children’s tooth brushing behaviour*** (Cronbach’s alpha = 0.633)					
**Factor TB1: Importance and intention to brush the child’s teeth** (Cronbach’s alpha = 0.618)					
b1	As a family, we intend on brushing our child’s teeth for him/her	positive	99.3	1.12	4.07
b2	We intend brushing our child’s teeth for him/her twice a day	positive	99.0	1.21	2.48
b3	The people in my family would feel it was important to help brush our child’s teeth twice a day	positive	99.3	1.35	1.48
b4	The people we know well would feel it was important to brush our child’s teeth twice a day	positive	86.1	1.65	0.90
b5	We feel able to brush our child’s teeth for him/her	positive	99.3	2.70	0.17
**Factor TB2: Parental efficacy in relation to child tooth brushing** (Cronbach’s alpha = 0.697)					
b6	If our child does not want to brush his/her teeth every day, we don’t feel we should make them	negative	97.4	3.40	−1.21
b7	I don’t know how to brush my child’s teeth properly	negative	96.4	3.28	−0.83
b8	It would not make any difference to our child getting tooth decay if we helped him/her brush every day	negative	93.4	3.29	−1.03
b9	We don’t have time to help brush our child’s teeth twice a day	negative	98.0	3.60	−1.73
b10	We cannot make our child brush his/her teeth twice a day	negative	94.4	3.39	−1.17
b11	It is not worth it to battle with our child to brush his/her teeth twice a day	negative	96.4	3.50	−1.61
**Factor TB3: Attitudes towards prevention** (Cronbach’s alpha = 0.588)					
b12	It is important to clean my child’s teeth every day so my child has a nice smile	positive	96.4	1.38	1.72
b13	If we brush our child’s teeth twice a day, we can prevent our child getting tooth decay in the future	positive	97.4	1.33	1.78
b14	If our child uses fluoride toothpaste, it will prevent tooth decay	positive	80.8	1.86	0.47
***Dimension SS: Parental attitudes towards child sugar snacking*** (Cronbach’s alpha = 0.689)					
**Factor SS1: Importance and intention to control child sugar snacking** (Cronbach’s alpha = 0.767)					
s1	We can prevent tooth decay in our children by reducing sugary foods and drinks between meals	positive	98.7	1.52	1.21
s2	As a family, we intend controlling how often our child has sugary foods or drinks between meals	positive	99.0	1.71	0.80
s3	The people in my family would feel it was important to control how often our child has sugary foods and drinks between meals	positive	98.0	1.61	1.04
s4	Our child eating sugary foods and drinks in between meals would cause tooth decay	positive	96.4	1.69	0.93
s5	The people we know well would feel it was important to control how often our child has sugary foods and drinks	positive	83.4	1.95	0.44
**Factor SS2: Parental efficacy in relation to controlling child sugar snacking** (Cronbach’s alpha = 0.677)					
s6	As a family, we feel it is difficult for us to stop our child having sugary foods	negative	97.0	2.86	−0.20
s7	It is worthwhile to give our child sweets/biscuits to behave well	negative	95.4	3.27	−0.85
s8	In our family, it would be unfair not to give sweets to our child every day	negative	95.4	2.63	0.07
s9	It is often too stressful to say no to my child when he/she wants sweets	negative	93.0	3.03	−0.51
***Dimension DD: Parental attitudes towards child dental decay*** (Cronbach’s alpha = 0.690)					
**Factor DD1: Perceived seriousness of tooth decay in children** (Cronbach’s alpha = 0.718)					
d1	As a family, we are confident we can reduce the chances of our child from getting tooth decay	positive	93.7	1.78	0.56
d2	Tooth decay will not get better by itself	positive	90.4	1.44	1.81
d3	Tooth decay would have major consequences on our child’s general health	positive	92.1	1.39	1.75
d4	Tooth decay is a serious problem in baby teeth	positive	91.7	1.40	1.05
d5	As parents, it is our responsibility to prevent our child getting tooth decay	positive	98.0	1.23	1.85
d6	Our child losing a baby tooth due to tooth decay would be upsetting	positive	93.0	1.44	1.25
d7	We feel it is important that we check our child’s teeth for decay	positive	98.4	1.19	2.54
**Factor DD2: Chance control—decay occurs by chance** (Cronbach’s alpha = 0.731)					
d8	No matter what we do, our child is likely to get tooth decay	negative	77.2	2.77	−0.07
d9	It is just bad luck if our child gets tooth decay	negative	84.8	3.38	−0.89
d10	If our child gets tooth decay, it is by chance	negative	85.8	3.48	−1.13
d11	Tooth decay runs in families	negative	83.8	2.48	0.53
d12	Some people just naturally have soft teeth	negative	88.7	2.14	0.66
**Factor DD3 External control—preventing decay is the dentist’s** **responsibility** (Cronbach’s alpha = 0.628)					
d13	It is the responsibility of the dentist to prevent our child getting tooth decay	negative	92.7	2.55	0.18
d14	Bringing our child to the dentist on a regular basis is the best way to prevent tooth decay	negative	96.7	1.79	0.92
d15	The dentist is the best person to prevent tooth decay in our child	negative	93.7	2.81	−0.19

Notes: ^1^ Agreeing with the statement expressed either a ‘positive’ or ‘negative’ attitude; ^2^ Percentage of answers ‘strongly agree’, ‘agree’, ‘disagree’ and ‘strongly disagree’. ^3^ Mean of answer codes: 1 = ‘strongly agree’, 2 = ‘agree’, 2.5 = ‘don’t know’ or missing answer, 3 = ‘disagree’, 4 = ‘strongly disagree’.

**Table 3:** Factorial structure of dimensions: results from exploratory factor analysis.

Dimensions and Items	Factors and Loadings		
**Dimension TB**	**TB1**	**TB2**	**TB3**
b1			0.527
b2			0.775
b3			0.345
b4			0.544
b5	−0.170		0.155
b6	0.764		
b7	0.432		
b8	0.406	0.427	
b9	0.517		
b10	0.785		
b11	0.748		
b12		0.653	
b13		0.759	
b14		0.678	
**Dimension SS**	**SS1**	**SS2**	
s1	0.688		
s2	0.698		
s3	0.768		
s4	0.795		
s5	0.628		
s6		0.624	
s7		0.741	
s8		0.660	
s9		0.793	
**Dimension DD**	**DD1**	**DD2**	**DD3**
d1	0.438		
d2	0.466		
d3	0.756		
d4	0.744		
d5	0.696		
d6	0.618		
d7	0.517		
d8		0.649	
d9		0.634	
d10		0.655	
d11		0.762	
d12		0.665	
d13			0.662
d14			0.698
d15			0.838

Notes: Dimension TB: Parental attitudes towards child tooth brushing behaviour; Dimension SS: Parental attitudes towards child sugar snacking; Dimension DD: Parental attitudes towards child dental decay.

**Table 4:** Results from multiple-group factor analysis and analogous estimates from the sum score analysis.

Groups of Respondents or Children to Be Compared	Multiple-Group Factor Analysis ^1^						Sum Score Analysis					
	Conditional Factor Means (Δχ^2^ (df); *p*) ^2^			Correlations between Factors (Δχ^2^; *p*) ^3^			Means of Sum Scores			Correlations between Sum Scores		
	TB	SS	DD	TB–SS	SS–DD	TB–DD	TB	SS	DD	TB–SS	SS–DD	TB–DD
Gender of respondents:	(Δχ^2^ = 0.687 (3); *p* = 0.876)			(Δχ^2^ = 23.416 (6); *p* = 0.001)								
males	0	0	0	0.218	0.277	0.820 *	27.89	22.19	21.24	0.261	0.158	0.557 ***
females	0.016	−0.001	0.060	0.683 *	0.781 ***	0.627 *	27.85	22.42	21.43	0.330 ***	0.337 ***	0.370 ***
Age of respondents:	(Δχ^2^ = 2.532 (3); *p* = 0.469)			(Δχ^2^ = 8.744 (6); *p* = 0.188)								
<35 years	0.027	0.023	−0.039	0.639 **	0.642 ***	0.715 **	28.06	22.52	21.38	0.331 ***	0.363 ***	0.363 ***
≥35 years	0	0	0	0.647 **	0.724 ***	0.854 **	27.60	22.23	21.43	0.300 ***	0.233 **	0.444 ***
Gender of children:	(Δχ^2^ = 2.954 (3); *p* = 0.399)			(Δχ^2^ = 10.757 (6); *p* = 0.096)								
boys	0	0	0	0.421 *	0.846 ***	0.838 ***	27.70	22.57	21.11	0.212 **	0.351 ***	0.444 ***
girls	−0.023	0.027	−0.058	0.622 **	0.729 ***	0.846 ***	27.98	22.24	21.65	0.449 ***	0.278 ***	0.339 ***
Age of children:	(Δχ^2^ = 1.942; *p* = 0.585)			(Δχ^2^ = 9.129; *p* = 0.166)								
<5 years	0.024	0.058	0.052	0.538 *	0.966 ***	0.682 **	27.94	22.72	21.55	0.348 ***	0.422 ***	0.411 ***
≥5 years	0	0	0	0.740 *	0.504 **	0.955 **	27.76	22.06	21.25	0.291 ***	0.198 **	0.378 ***
Children from kindergartens located in:	(Δχ^2^ = 6.553; *p* = 0.088)			(Δχ^2^ = 15.131; *p* = 0.019)								
urban area	0.060	0.135 *	0.092	0.361 *	0.590 ***	0.699 **	28.19 **	22.77 **	21.67 *	0.242 ***	0.254 ***	0.327 ***
rural area	0	0	0	0.508 **	0.632 ***	0.793 **	27.18	21.62	20.87	0.414 ***	0.368 ***	0.464 ***
Education level of respondents:	(Δχ^2^ = 13.856 (3); *p* = 0.003)			(Δχ^2^ = 13.072 (6); *p* = 0.042)								
less than college or university	0	0	0	0.494 **	0.588 ***	0.733 ***	27.82	22.30	20.47	0.339 **	0.363 ***	0.510 ***
college or university	−0.055	0.010	0.137 **	0.380 *	0.522 ***	0.656 **	27.87	22.42	21.72 ***	0.314 ***	0.299 ***	0.359 ***
The respondent brushes his/her teeth:	(Δχ^2^ = 5.708 (3); *p* = 0.127)			(Δχ^2^ = 4.458 (6); *p* = 0.615)								
2 times a day	0.071	0.070	0.132 *	0.658 **	0.756 ***	0.846 ***	28.32 ***	22.51	21.62 *	0.330 ***	0.327 ***	0.373 ***
less than 2 times a day	0	0	0	0.868 **	0.912 **	0.898 **	26.43	22.01	20.72	0.269*	0.251 *	0.377 ***
The child brushes his/her teeth:	(Δχ^2^ = 34.184 (3); *p* < 0.001)			(Δχ^2^ = 3.372 (6); *p* = 0.761)								
2 times a day	0.357 ***	0.127 *	0.023	0.526 **	0.767 ***	0.786 ***	28.81 ***	22.74*	21.49	0.299 ***	0.357 ***	0.373 ***
less than 2 times a day	0	0	0	0.899 **	0.727 ***	0.828 ***	26.67	21.96	21.29	0.304	0.246 **	0.448 ***
How does the child brush his/her teeth:	(Δχ^2^ = 18.115 (3); *p* < 0.001)			(Δχ^2^ = 9.340 (6); *p* = 0.135)								
the child brushes his teeth by himself/	0	0	0	0.713 *	0.862 ***	0.624 *	27.45	21.98	20.72	0.284 **	0.363 ***	0.307 ***
parents help the child brush his teeth	0.011	0.119*	0.171 ***	0.574 *	0.670 ***	0.930 **	28.21 *	22.75 *	22.01 ***	0.337 ***	0.219 **	0.462 ***
The child visits the dentist:	(Δχ^2^ = 9.216 (3); *p* = 0.027)			(Δχ^2^ = 2.030 (6); *p* = 0.917)								
once a year or more frequently	0.049	0.103 *	0.134 **	0.693 **	0.653 ***	0.824 **	28.19 *	22.68 *	21.79 ***	0.349 ***	0.311 ***	0.367 ***
irregularly	0	0	0	0.604 *	0.888 ***	0.858 *	27.31	21.82	20.78	0.241 **	0.279 **	0.400 ***
Respondent’s dental health status:	(Δχ^2^ = 9.200 (3); *p* = 0.027)			(Δχ^2^ = 5.318; *p* = 0.504)								
good	0.060	0.005	0.185 **	0.638 **	0.708 ***	0.795 **	28.10 **	22.43	21.65 ***	0.338 ***	0.331 ***	0.372 ***
poor	0	0	0	0.658 **	0.738 ***	0.824 **	26.82	22.22	20.36	0.233	0.239	0.391 **
Child’s dental health status:	(Δχ^2^ = 2.994; *p* = 0.393)			(Δχ^2^ = 6.443; *p* = 0.375)								
good	0.028	0.040	0.116	0.622 *	0.716 ***	0.839 *	28.08 *	22.48	21.61 **	0.326 ***	0.333 ***	0.349 ***
poor	0	0	0	0.685	0.883 *	0.625	26.98	22.06	20.59	0.287 *	0.222	0.476 ***

Notes: ^1^ Characteristics were estimated when the model was constrained for equal factor loadings and intercepts in the equation for predicting observed variables between groups; ^2^ The change in chi-square when introducing the equal-factor-means constraint; ^3^ The change in chi-square when introducing the equal-factor-covariations constraint; * *p* < 0.05, ** *p* < 0.01, *** *p* < 0.001 (tests for equal means or zero correlation).

## Data Availability

The data set is available on request from the corresponding author. The data are not publicly available due to privacy restrictions and intellectual property protection.
